# Self-aligning roly-poly RFID tag

**DOI:** 10.1038/s41598-022-06061-6

**Published:** 2022-02-08

**Authors:** Dmitry Dobrykh, Ildar Yusupov, Pavel Ginzburg, Alexey Slobozhanyuk, Dmitry Filonov

**Affiliations:** 1 School of Electrical Engineering, Tel Aviv University, 69978 Tel Aviv, Israel; 2 School of Physics and Engineering, ITMO University, 197101 Saint Petersburg, Russia; 3 Center for Photonics and 2D Materials, Moscow Institute of Physics and Technology, 141700 Dolgoprudny, Russia

**Keywords:** Electrical and electronic engineering, Mechanical engineering

## Abstract

Radio frequency identification (RFID) is a mature technology that allows contactless data readout via a wireless communication link. While numerous passive RFID tags are available on the market, accurate alignment between tags and readers is required in a vast majority of cases to mitigate polarization mismatches. We show that enhancing electromagnetic designs with additional mechanical degrees of freedom allows bypassing fundamental limitations and approach ideal performances. Here, we demonstrate a new miniature tag, accessible from any direction and immune to rotations in space. Our tag is made of a high permittivity ceramic resonator, inductively coupled to a metal ring, which contains an RFID chip. The structure is placed inside a spherical plastic holder. In this architecture, the ceramic resonator serves several functions. First, it allows reducing the device footprint without significant bandwidth degradation. Second, it acts as a bob, aligning the electromagnetic structure parallel to the ground, regardless of its initial orientation in space. The bob is designed to slide inside the plastic holder. This roly-poly effect relaxes the constraint on a mutual tag-reader orientation, including the polarization mismatch, and provides next to perfect long-range operation. Being only 55 mm in diameter, our device can be interrogated from a 12 m distance, regardless of the tag’s orientation in space. Introducing mechanical degrees of freedom into electromagnetic designs allows obtaining new functionalities, contributing to applications where a mutual orientation between transvers is required.

## Introduction

Radio frequency identification (RFID) is a widely spread technology used for a wireless data exchange^1^. An RFID system typically consists of a reader device that interrogates an information holder—a tag. Many applications demand extremely low-cost consumable tags, which forces implementing the latter as a simple passive system, consisting of an integrated circuit (IC) and an antenna for providing a wireless communication. Hereinafter, we will concentrate on passive RFID architecture (e.g., EPCGEN2 protocol), which is among the most widely spread nowadays. Passive RFID is based on time-modulated backscattering. A reader initiates a communication with a passive tag by transmitting an electromagnetic wave. Electromagnetic energy harvested by the tag’s antenna is partially rectified to power an IC, modulating the system’s impedance in time. The information written on the IC is then imprinted on a time-modulated backscattered signal, which is decoded by the reader’s electronics. Owing to its high efficiency and low cost, RFID is employed in a broad range of practical applications, including warehouses and logistics, billing systems, biometric identifiers, healthcare monitoring, and many other areas.

One among prospective future applications of RFID is the Internet of Things (IoT). In this paradigm, various devices communicate with each other and with a surrounding environment without a need for human-to-human or human-to-computer interactions^2,3^. As the next step in this promising direction, one can envision the Internet of *Small* Things (IoST) concept, where each product (even miniature, low-cost items) has a unique identification number, assessable by a reader. This is exactly the point where passive RFID will start playing a key role. However, tight requirements are then set on the tag’s characteristics. In particular, low cost, small footprint, large reading distance, and omnidirectional response from any direction in space are required. Unfortunately, standard commercial realizations typically combine a pair of those functionalities simultaneously for the most. One of the main problems of existent implementations is a polarization mismatch, which emerges due to the incorrect positioning of a reader antenna relative to the tag. In other words, to perform a data transfer, a reader’s antenna and a tag should be aligned in respect to each other. This requirement is hard to meet on multiple occasions, such as an airport luggage conveyor, where bags can obtain any accidental orientation, or an autonomous collection of RFID-labeled rubbish by a robot cleaner, as part of the IoST paradigm.

The requirement of a certain mutual reader-tag orientation in space can significantly limit the reading performance due to the certain ‘dead’ sectors, from which the tags cannot be interrogated. Several designs to achieve omnidirectional operation have been demonstrated^4–9^, but they still cannot fit all of the beforehand mentioned requirements, which reduces the number of potential applications.

Another important parameter of a passive RFID tag is its size. Footprint miniaturization is crucially important for the Internet of Small Things applications. There are several common techniques for the tag’s size reduction, including antenna meandering^10^, fractal antennas^11,12^, using dielectric substrates^13,14^, and dielectric resonator antennas^15–17^. Though, it is worth noting several restrictions on omnidirectional operation, set by fundamental laws of electromagnetism. In particular, obtaining an ideal omnidirectional linearly polarized radiation pattern is fundamentally impossible, e.g.,^18–20^. However, those limitations do not apply if non-electromagnetic degrees of freedom are introduced.

**Figure 1:**
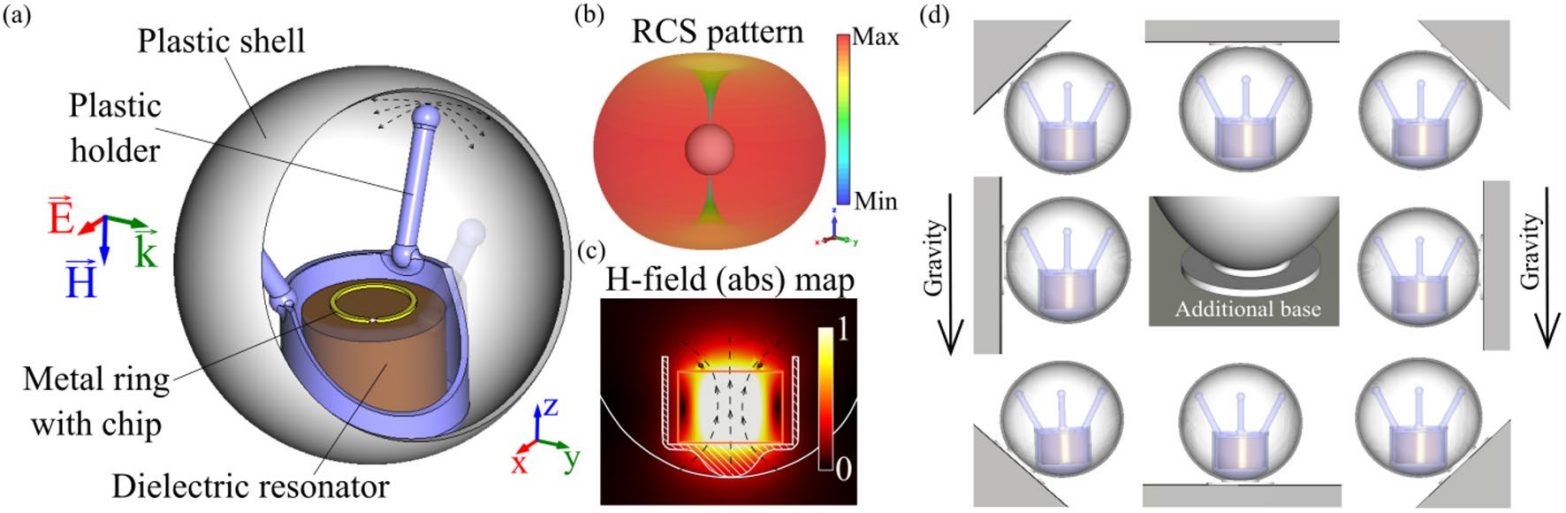
(**a**) Roly-poly RFID tag. (**b**) 3D radiation pattern of the tag, alighted parallel to the ground. (**c**) The magnetic field amplitude distribution at the cut plane along the cylinder’s axis. The arrows show the H-field direction. (**d**) Mechanical operational principle. The tag is attached to a surface (grey polygons) with a sticker. Regardless the orientation of the surface, the tag is self-alighted parallel to the ground.

Here, we propose a new concept of a ceramic RFID tag, insensitive to rotations in space. This property is achieved by introducing additional mechanical degrees of freedom within the design. Our architecture is based on a high-index ceramic resonator encapsulated in a plastic enclosure. The ceramic elements serve two essential functions. First, they act as a dielectric resonant antenna (DRA)^21,22^. DRAs utilize high-permittivity materials for achieving device miniaturization without significant bandwidth degradation and other purposes^23,24^. The second function of ceramic elements is mechanical. The resonator is free to rotate inside the enclosure in all three directions. Having a displaced center of mass and being subject to gravitation, the resonator will always be aligned parallel to the earth, regardless of the physical orientation of the enclosure. This roly-poly behavior solves the polarization mismatch issues, while the high-index compact resonator allows approaching miniaturized design. The general principle of the tag’s operation is shown in Fig. 1.

The operation principle of the device is as follows: a ceramic cylinder, supporting a magnetic dipole mode, is functionalized with a miniature non-resonant metal split ring. A standard RFID chip is soldered in the ring’s gap and placed on top of the dielectric cylinder. Near-field coupling allows converting the displacement current of the resonator into the conduction currents in the ring, activating the chip. A dielectric resonator with a metallic ring is then placed in the plastic holder. The holder has three posts, touching a spherical enclosure. The fourth contact point is the bottom of the holder. The structure is free to slide within the spherical enclosure, in which the inner surface is lubricated to reduce friction. The dielectric resonator is made of high-quality ceramic, which mass dictates the mechanical dynamics. The center of mass of the structure is aligned so that the cylinder approaches the lowest point within the enclosure. Plastic elements used in the design are transparent to electromagnetic waves. The roly-poly operation ensures the alignment of the split ring parallel to the earth. If the reader’s antenna is set to have magnetic field polarization perpendicular to the ground, the interrogation does not affected by polarization mismatch issues. In order to attach the spherical enclosure to a flat object, subject to labeling, an additional sticky element can be implemented on the outer surface of the enclosure. Furthermore, the outer shape can be made arbitrary, as it does not affect the device operation. Figure 1d shows the mechanical self-alignment of the device. Grey polygons demonstrate an arbitrary oriented object, labeled with the tag. The resonator is always alighted parallel to the ground owing to the gravitational force.

## Ceramic tag size reduction

A footprint of RFID tag is governed by its antenna. The miniaturization of the structure can be performed by utilizing dielectric resonators since the wavelength within a material scale with the refractive index. Consequently, the size reduction can be compensated with the permittivity increase. However, this approach comes at a price—the operational bandwidth and the antenna gain are degraded. While the first aspect is less important for RFID applications (a single channel in the communication protocol is 250–500 kHz), the tag’s antenna gain significantly affects the reading range.

To assess the proposed miniaturization technique, we performed a numerical simulation in CST Microwave Studio. The range of resonator’s permittivity was taken to vary from 100 to 700, which complies with the fabrication capabilities of our vendor^25^. The tag’s reading range is calculated with the Friis model:^26,27^

1 $$L=\frac{\lambda }{4\pi }\sqrt{\frac{{P}_{t}{G}_{TR}{G}_{t}\tau }{{P}_{ch}}} ,$$

where ${P}_{t}$ is the power transmitted by the reader, ${G}_{TR}$ is the gain of the reader Tx/Rx antenna, ${G}_{t}$ is the gain of the tag’s antenna, $\tau$ is the power transmission coefficient (matching parameter), ${P}_{ch}$ is the IC’s sensitivity, and $\lambda$ is the operational wavelength. The transmission coefficient $\tau$ in numerical simulations was calculated as ($1-{\left|{S}_{11}\right|}^{2})$.

It should be noted that the Friis equation evaluates only the downlink part of the communication, i.e., reader to tag. Here, the IC’s sensitivity (${P}_{ch}$) is the limiting factor. The second part of the communication is the uplink—tag transmission to the reader. Here, the limiting factor is the reader’s sensitivity. The reading distance of the entire system is governed by the shortest one among the two. However, in the case of state-of-the-art equipment, downlink limits the reading distance for passive tags. Hence, Eq. (1) is sufficient for making the assessment.

**Figure 2:**
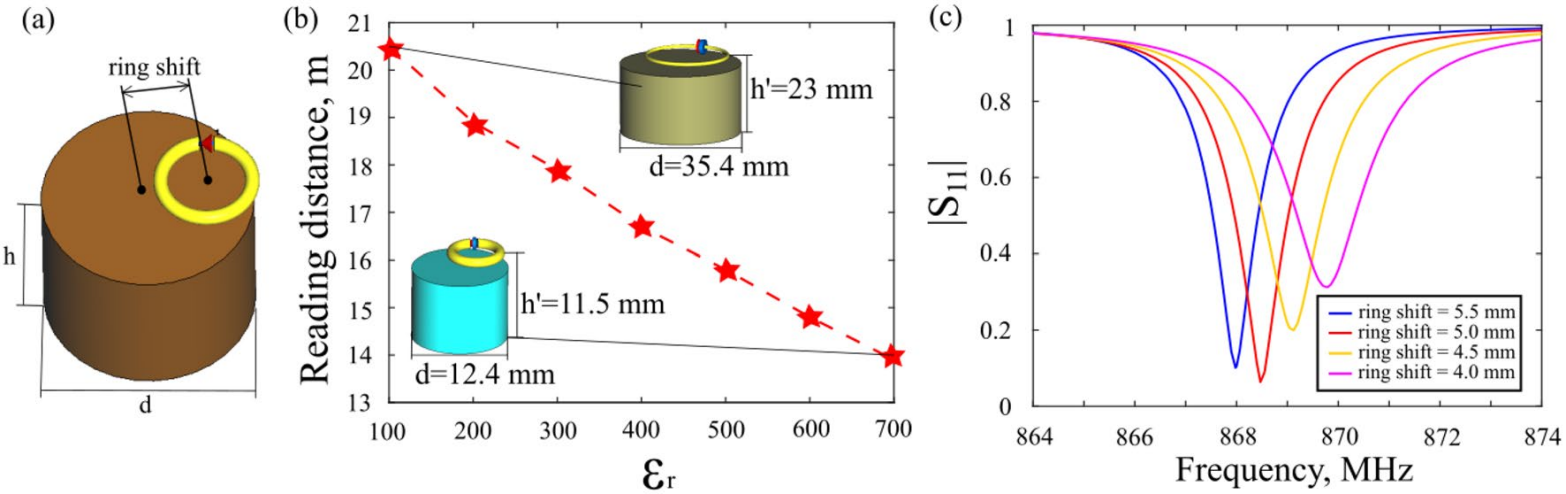
(**a**) Ceramic tag scheme. (**b**) Numerical assessment of the ceramic tags reading distance as a function of the cylinder’s permittivity. The operational frequency is constant *f*_*0*_ = 867 MHz. The insets show the structures and their dimensions for two points on the graph. (**c**) Numerical |S_11_| spectra of the ceramic tag in free space for several ring’s positions (indicated in captions).

The geometry of the tag appears in Fig. 1a. Figure 2b demonstrates the tag’s reading distance as the function of the cylinder’s permittivity. The parameters of the dielectric resonator at the first calculation point are as follows: radius $R=17.7$ mm, height $h=21$ mm, dielectric permittivity ${\varepsilon }_{r}=100$ with $tan \delta =4\times 1{0}^{-4}$ (see insert in Fig. 2b). Such parameters allow operation at 865–868 MHz which is Europe EPCGEN2 UHF RFID frequency standard. The radius of the nonresonant metal split ring is 10.6 mm. The ring’s radius was taken as $0.6\cdot R$ at the entire calculations.

To optimize the matching conditions between the chip and the tag’s antenna, we made a numerical analysis with a discrete port with a complex impedance $Z=14.1-j148.6$ Ω, taken from the vendor’s IC’s datasheet (Impinj Monza R6). Numerical optimization over cylinder’s parameters (radius, height, permittivity), ring’s parameters (radius, strip thickness), and mutual orientation between the cylinder and the ring was performed. The impedance matching was achieved for 865–867 MHz band by optimizing the resonator’s radius and height, ring’s radius and its position with respect to the cylinder. The mutual location between the ring and the cylinder (the distance above the surface and off-axis displacement) affects the inductive coupling. This parameter is directly mapped on the impedance matching conditions. Controlling the relative geometry allows obtaining the matching and, furthermore, tuning the operational frequency. Figure 2c shows the matching curves (S_11_ spectra) for several ring’s displacements. The displacements path is shown in Fig. 2a. It is worth noting that in the subsequent experimental studies the impedance matching can be tuned my introducing slight mechanical displacements manually. This approach significantly simplifies the experiment, which has to be performed at exact pre-defined frequencies.

The next step is the resonator’s miniaturization and the related performance assessment. For this purpose, the permittivity of the resonator was gradually increased, and the resonant shift was compensated by tuning the cylinder’s height (h) and radius (r). As a result, magnetic dipolar resonance remained at 867 MHz. The ring’s location was also adjusted to maintain high impedance matching $(\tau \ge 0.96)$. The results of the calculations appear as red stars in Fig. 2b, while the dashed line is a spline interpolation. The losses of the ceramic resonator were taken as $tan \delta =4\times 1{0}^{-4}$ over the entire range, based on the data from our vendor Ltd “Ceramics”^25^. The chip’s sensitivity was taken as -20 dBm (corresponding to the Impinj Monza R6 chip), the reader’s antenna gain, and the accepted power were taken as 7.5 dB and 27 dBm, according to our experimental setup. Table 1 contains the parameters that were obtained in numerical simulations.

**Table 1:** Numerically calculated parameters of the ceramic tag with different values of dielectric permittivity.

${\varepsilon }_{r}$	Gain $({G}_{t})$	Matching parameter ($\tau$)	Reading distance (m)	Resonator’s in-plane area (mm^2^)
100	1.44	0.97	20.5	4303
200	1.22	0.98	18.9	2450
300	1.12	0.96	17.9	1343
400	0.97	0.96	16.7	1048
500	0.86	0.98	15.8	831
600	0.76	0.96	14.8	703
700	0.67	0.96	13.9	611

The results in Fig. 2b show a linear drop of the reading range with the permittivity increase. The benefit, however, comes as the size reduction. The resonator’s area is reduced by a factor of seven, which complies with the dielectric permittivity change (from 100 to 700), while the reading range is reduced only by 1.5 times. The bandwidth (− 10 dB level) decreased from 3 to 0.45 MHz in the calculated permittivity range. The limitation for the tag antenna reduction is the bandwidth. One frequency channel occupies 250 kHz for Europe UHF RFID band (865–868 MHz) and 500 kHz for US UHF RFID band (902–928 MHz). Note that our design makes a non-uniform scaling in the cylinder’s radius and height. The results obtained show a more complex size reduction behaviour rather than just a uniform shrinkage with the refractive index. A mere approximation for the high-index open cylindrical resonator can be obtained by assuming close perfect electric conductor boundaries, e.g.,^28^.

## Roly-poly holder

**Figure 3:**
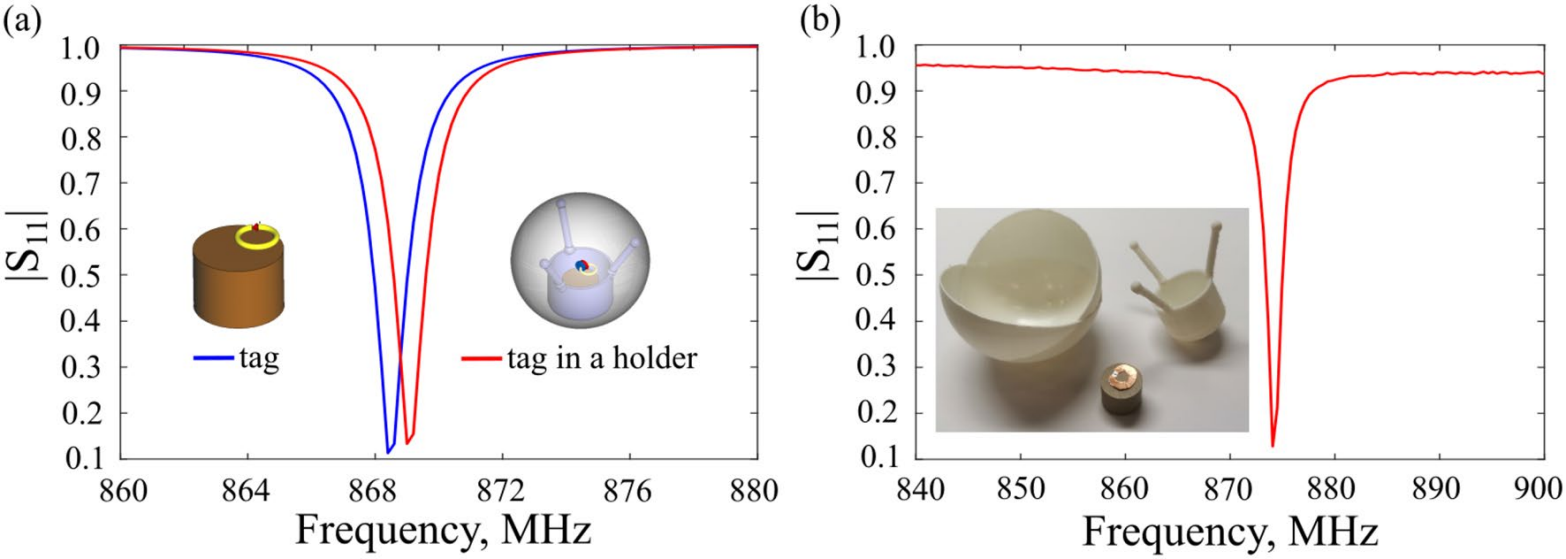
(**a**) Numerical |S_11_| spectra of the ceramic tag in free space and within the roly-poly holder. The impedance of the port is equal to the value provided by the vendor (Impinj Monza R6). The inset shows the structures. (**b**) Experimental |S_11_| spectrum of the fabricated ceramic resonator, measured by a small magnetic probe. The inset shows the components of the proposed RFID tag.

To demonstrate a negligible influence of the holder on the electromagnetic properties of the tag, the following numerical analysis was performed. The parameters of the resonator were chosen to be r = 7.25 mm, h = 11 mm, ${\varepsilon }_{r}=506$ with $tan \delta =4\times 1{0}^{-4}$ (the permittivity was retrieved at 1 GHz by our vendor). Such parameters allow manufacturing a compact RFID tag with a reading range over 15 m (see Table 1). The numerical port with $Z=14.1-j148.6$ Ω impedance was plugged within the ring’s gap, and the complex reflection (|S_11_| parameters) spectra were numerically calculated for the tag in free space and the tag within the plastic (${\varepsilon }_{r}$ = 3) holder, placed into the shell (Fig. 3a). The diameter of the shell is d = 55 mm. The parameters of the plastic were taken from^29^.

The results show almost the same impedance matching in both configurations with only a 1 MHz spectral shift. The parameters of the holder will be discussed in the experimental section. The analysis here shows that the mechanical design can be performed almost independently from the electromagnetic. A slight adjustment of the ring’s position regarding the ceramic resonator can be made to compensate for the spectral shift produced by the surrounding plastic materials.

## Experimental demonstration

To demonstrate the real performance of the proposed architecture, we conducted a long-range readout experiment in the anechoic chamber. An experimental sample was fabricated based on a dielectric resonator with radius = 7.25 mm, height $h=11$ mm, and dielectric permittivity ${\varepsilon }_{r}=506.$ The resonant frequency of the cylinder was measured by a small magnetic probe (Fig. 3b). After that nonresonant copper ring with an RFID chip Impinj Monza R6 was placed on the top of the resonator. By changing the position of the ring, the tag was tuned to operate at 867 MHz.

At the next step, the tag was placed in a plastic holder made of ABS plastic. The structure had three posts (see Fig. 1a), which were fabricated with the aid of 3D printing (model Raise3d Pro2). As for the enclosing shell, we used a cellulose ping-pong ball with a diameter of 55 mm. Its inner surface was lubricated with silicone grease to reduce friction. The overall weight of the entire structure was 17 g.

**Figure 4:**
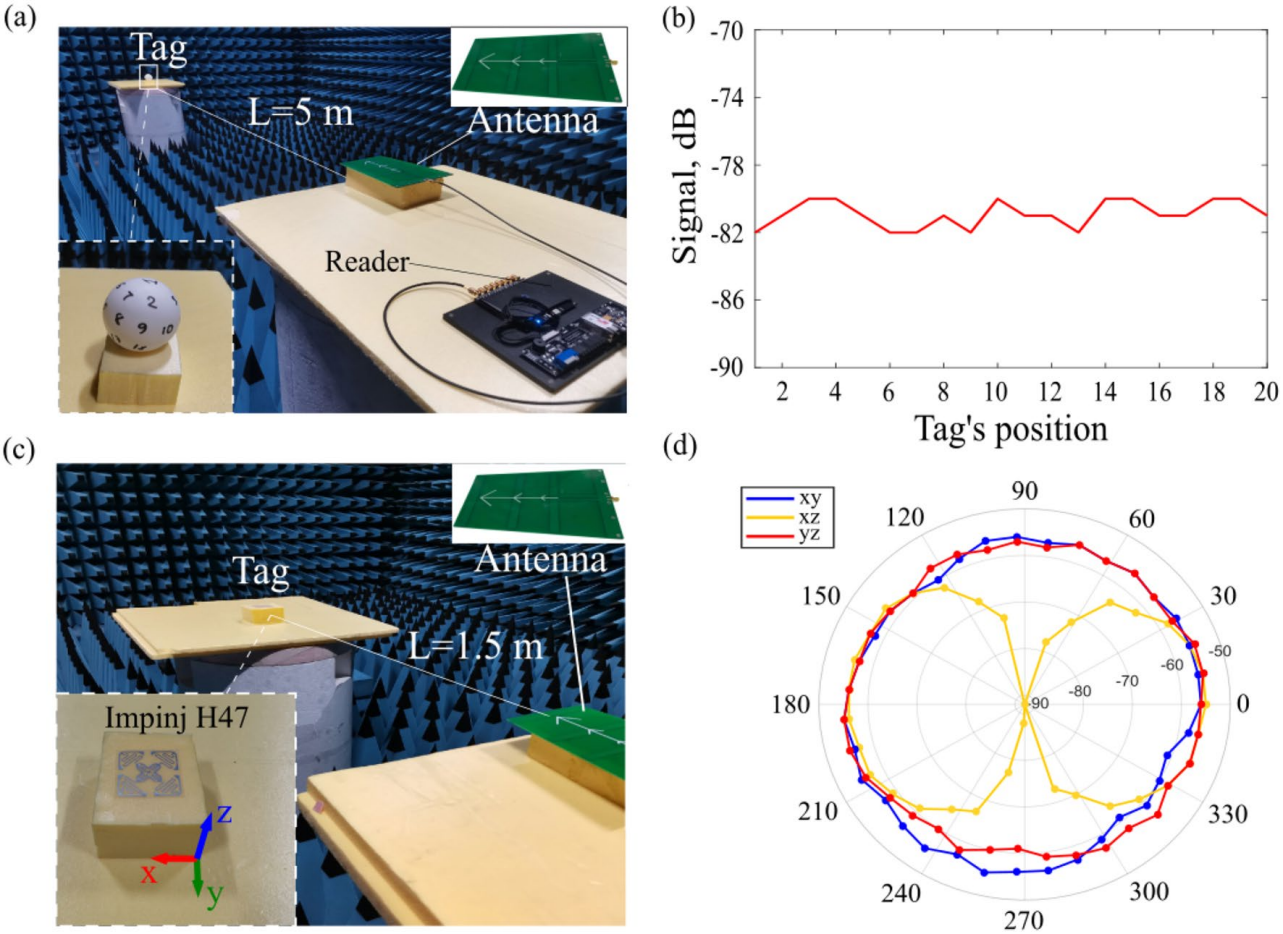
(**a**) Photo of the experimental setup with our tag in an anechoic chamber. (**b**) The received signal power as a function of the tag’s orientation in space (marks are in the inset to panel (**a**)). (**c**) Photo of the experimental setup with Impinj H47 tag in an anechoic chamber. (**d**) Angular diagram of the received signal power in xy, xz and yz coordinate planes.

The tag’s performance was evaluated in a readout configuration (the photograph of the experiment layout is shown in Fig. 4a). A commercial reader device (Impinj R2000 module) was used, and the amplitude of the received signal was monitored. As the reader’s antenna, we used a printed Yagi-Uda antenna (four directors), with − 13 dB matching in the 850–900 MHz frequency range (see Fig. 4a). The gain of the antenna was estimated to be 7.5 dBi at a frequency of 867 MHz. The transmitted power (${P}_{t}$) was set to 27 dBm, which does not exceed 2 W of the Equivalent Radiated Power owing to the international regulations for Europe UHF RIFD band (the reader’s antenna gain is 7.5 dB + 27 dB power − 2.15 dB < 33 dB restricted).

The tag was placed at an L = 5 m distance from the reader, and immunity to the mutual orientation was checked. For this purpose, 20 equally spaced points on the spherical shell were chosen and marked—see the inset in Fig. 4a. The amplitude of the received signal was monitored for each position. Figure 4b demonstrates the amplitude of the received signal as a function of the tag’s orientation in space. It can be seen the signal level is almost insensitive to the tag’s rotation, verifying the roly-poly operation.

To further underline the advantage of our design, we compared performances with the dual-dipole RFID tag Impinj H47, which is widely used for compensating polarization mismatch (Fig. 4c). This tag was assessed in the same setup. Using 27 dB reader’s transmitted power, we placed the tag 1.5 m apart and monitored the received power at different mutual orientations. 3 rotating planes of the tag were checked. This dual-dipole tag allows outperforming a single dipole architecture in case of an interrogation with a linearly polarized antenna. However, Impinj H47 has dead zones, as it is seen in Fig. 4d. This behavior is expected as the tag has a planar geometry. It is worth noting again that there are solutions, based on volumetric tags, which have quasi-isotropic responses, e.g.^4–9^. However, those typically have a small reading rage, which does not exceed 2–3 m. Our roly-poly tag encompasses both small footprint, long reading rang and all angle accessibility.

It should be noted that a 5 m demonstrated reading distance (which is the largest dimension of our anechoic chamber) is not the maximal reading range of the fabricated tag. An additional measurement in a spacious auditorium demonstrated a 12 m reading distance. Further extension of the reading range is limited by the tag’s sensitivity. This number could be increased up to 15 m with better impedance matching by iterative changing the copper ring’s radius and its relative position in respect to the cylinder. However, even with a 12 m reading range and with a 9500 mm^2^ footprint (in-plane), the device is well-positioned among state-of-the-art long-range RFID tags. A more detailed comparison of the current state-of-the-art long-range RFID passive tags can be found in^15,30^.

## Conclusion

The new type of a passive ceramic RFID tag, insensitive to rotations in space, was demonstrated. The architecture is based on a high-index dielectric resonator inductively coupled with a split-ring with an integrated RFID chip. This geometry is encapsulated in a plastic 3D printed holder, placed inside the thin plastic shell. The overall size of the proposed structure was miniaturized and had a spherical shape of 55 mm in diameter. A stable reading with no dependence on the tag’s spatial orientation position was experimentally demonstrated in long-range configuration, approaching 12 m interrogation distance. Small self-aligning RFID tags with a long reading range can be extremely useful in numerous applications, including airport baggage tracking, quality control of packed goods, Internet of *Small* Things, logistics, security, and many others.
